# Combined diabetic ketoacidosis and hyperosmolar hyperglycemic state in type 1 diabetes mellitus induced by immune checkpoint inhibitors: Underrecognized and underreported emergency in ICIs-DM

**DOI:** 10.3389/fendo.2022.1084441

**Published:** 2023-01-04

**Authors:** Wenjing Zhang, Jiexiu Chen, Juan Bi, Nan Ding, Xin Chen, Zhuo Wang, Yang Jiao

**Affiliations:** ^1^ Department of Pharmacy, Shanghai Changhai Hospital, the First Affiliated Hospital of Naval Medical University, Shanghai, China; ^2^ Department of Clinical Pharmacy, Sichuan Provincial Maternity and Child Health Care Hospital, Affiliated Women’s and Children’s Hospital of Chengdu Medical College, Chengdu Medical College, Chengdu, China; ^3^ Department of Pharmacy, Anhui Provincial Corps Hospital, Chinese Peoples Armed Police Force, Hefei, China; ^4^ Department of Respiratory and Critical Care Medicine, Shanghai Changhai Hospital, the First Affiliated Hospital of Naval Medical University, Shanghai, China

**Keywords:** immune checkpoint inhibitor, programmed cell death-1, hyperosmolar hyperglycemic state, diabetes mellitus, combined DKA-HHS

## Abstract

**Background:**

Combined diabetic ketoacidosis (DKA) and hyperosmolar hyperglycemic state (HHS) secondary to immune checkpoint inhibitors (ICIs) is extremely rarely reported among ICIs- diabetes mellitus (DM) cases and is always ignored by physicians. This study aimed to conduct a systematic review to recognize better the rare adverse event of combined DKA-HHS associated with immune checkpoints.

**Methods:**

A electronic search in Pubmed/Cochrane/Web of Science, complemented by manual searches in article references, was conducted to identify clinical features of ICIs-combined DKA-HHS.

**Results:**

we identified 106 patients with ICIs- type 1 diabetes mellitus (T1DM) from 82 publications: 9 patients presented a coexistence of metabolic acidosis, severe hyperglycemia, and/or DKA; All patients were not diagnosed as combined DKA-HHS. Compared with ICIs-DKA patients, combined DKA-HHS cases were prone to higher hyperglycemia (1020 ± 102.5 vs 686.7 ± 252.6mg/dL). Moreover, acute kidney injury (87.5% vs 28.6%) and prior chemotherapy (66.7% vs 31.6%) showed higher occurrences with the onset of ICIs-HHS or combined DKA-HHS.B

**Conclusions:**

Combined DKA-HHS portends a poor diagnosis in patients with coexistence features of DKA and HHS, which healthcare professionals and patients should be aware of due to differences in treatment. Our observational retrospective case series shows that patients with more risk factors were more likely to develop combined DKA-HHS. We are the first to report this group of patients’ clinical characteristics and outcomes.

## Introduction

Although various treatment methods, such as surgery, chemotherapy, and radiation therapy, have been used to treat various tumors, immune checkpoint inhibitors significantly promote tumor cell death as a new class of drugs. Immune checkpoint inhibitors, such as programmed cell death 1 (PD-1)/program death-ligand 1 (PD-L1) inhibitors and cytotoxic T-lymphocyte antigen 4 (CTLA-4) destroy tumor cells through T-cell-mediated mechanisms by reversing immune escape or evasion. An unfortunate side effect of ICIs treatment is the development of immune-related adverse events (irAEs) due to increased T-cell activation (1).

ICIs-DM, differ from other irAEs in that they are rare (as the incidence is estimated at ~1%) and potentially life-threatening (2–4). Most of them are reported as T1DM or fulminant type 1 diabetes, with half of the cases presenting with DKA (5, 6), while combined DKA-HHS are rarely reported than DKA.

The hyperosmolar hyperglycemic state is characterized by severe hyperglycemia and hyperosmolality without significant ketosis and acidosis. Interestingly, ICIs-induced combined DKA-HHS have combined features of hyperosmolality and metabolic acidosis, and/or DKA (7). In addition, unlike HHS of spontaneous DM that has been frequently reported in adult patients with type 2 DM (T2DM), ICIs-induced combined DKA-HHS was significantly sensitive to insulin therapy and reported in T1DM. Given the limited data available regarding the clinical characteristics and prognosis of patients presenting with combined features of metabolic acidosis, severe hyperglycemia, and/or DKA, there is no accepted definition to characterize this population. Therefore, it is essential for early recognition of patients who present with combined DKA-HHS.

Here we performed a systematic review and compared ICIs- induced combined DKA-HHS with ICIs-DKA cases to describe the clinical characteristics and predisposing factors, as well as the optimal treatment of ICIs- induced combined DKA-HHS. The findings of this study will provide a valuable reference for the recognition of ICIs- induced combined DKA-HHS.

## Methods

### Search strategy

We conducted a systematic review on PubMed, Web of Science, and Cochrane databases for case reports and series that reported new-onset diabetes after ICIs therapy published before March 2022. The comprehensive search used the terms “immune checkpoint inhibitor” and “diabetes” (details of the search strategy were shown in **Supplementary Material S Table 1**). Two authors took part in the whole screening process. References in the included reports were also reviewed for additional cases.

### Inclusion and exclusion criteria

The inclusion criteria included the following items: 1) case report included ICIs related medication information, blood glucose at admission, and other related laboratory values; 2) the reported case was diagnosed HHS or DKA in published papers or met the DKA/HHS and combined DKA-HHS criterion (8, 9); 3) the paper was reported in English. Conferences, reviews, clinical trials, and cases that had an undefined relationship with ICIs therapy or had insufficient information to diagnose DKA and HHS, were excluded. We extracted data regarding age, gender, ethnicity, cancer type, previous medical history of the patient, usage regimen and cycles of ICIs therapy, diabetes tests of glucose, arterial blood gas, glycated hemoglobin, blood or urine ketone, C-peptide, islet autoantibodies, and human leukocyte antigen (HLA) genotype. Other irAEs, suspected infection, kidney function, and exocrine pancreatic function were also documented.

Hyperosmolality was defined as an effective plasma osmolality ≥320 mOsm/kg[measured when available, and otherwise calculated 2*[measured serum Na+ (mEq/L)] + glucose (mg/dl)/18 referenced from ref (8)]. Case definition of hyperglycemic crises on admission was:

HHS: HHS include a plasma glucose level >33.3 mmol/l, serum osmolality>320 mmol/kg and no appreciable metabolic acidosis and ketonaemia (8, 9);

DKA: DKA comprises hyperglycaemia(blood glucose ≥200 mg/dL), hyperketonaemia and metabolic acidosis(pH ≤7.3 or bicarbonate ≤15 mEq/L) (8, 9)

Combined DKA-HHS: plasma osmolarity ≥ 320 mOsm/kg, initial blood sugar ≥ 600 mg/dL, pH ≤ 7.3, and ketonuria and/or ketonemia (9)

### Statistics

Qualitative and quantitative variables were respectively expressed as frequency (percentage), median with interquartile range (IQR) for abnormal distribution data, and mean with standard deviation for normal distribution data. Differences in qualitative variables between the HHS and DKA cases were assessed with χtwo tests or Fisher exact test. Student’s t-test or Mann-Whitney U test was used for quantitative ones as appropriate. Multivariate analysis for risk factors was unable to perform because of the small cases of HHS. A p-value <0.05 was considered statistically significant. All statistical analyses and data management were performed with SPSS (IBM, version 28).

## Results

**Figure 1:**
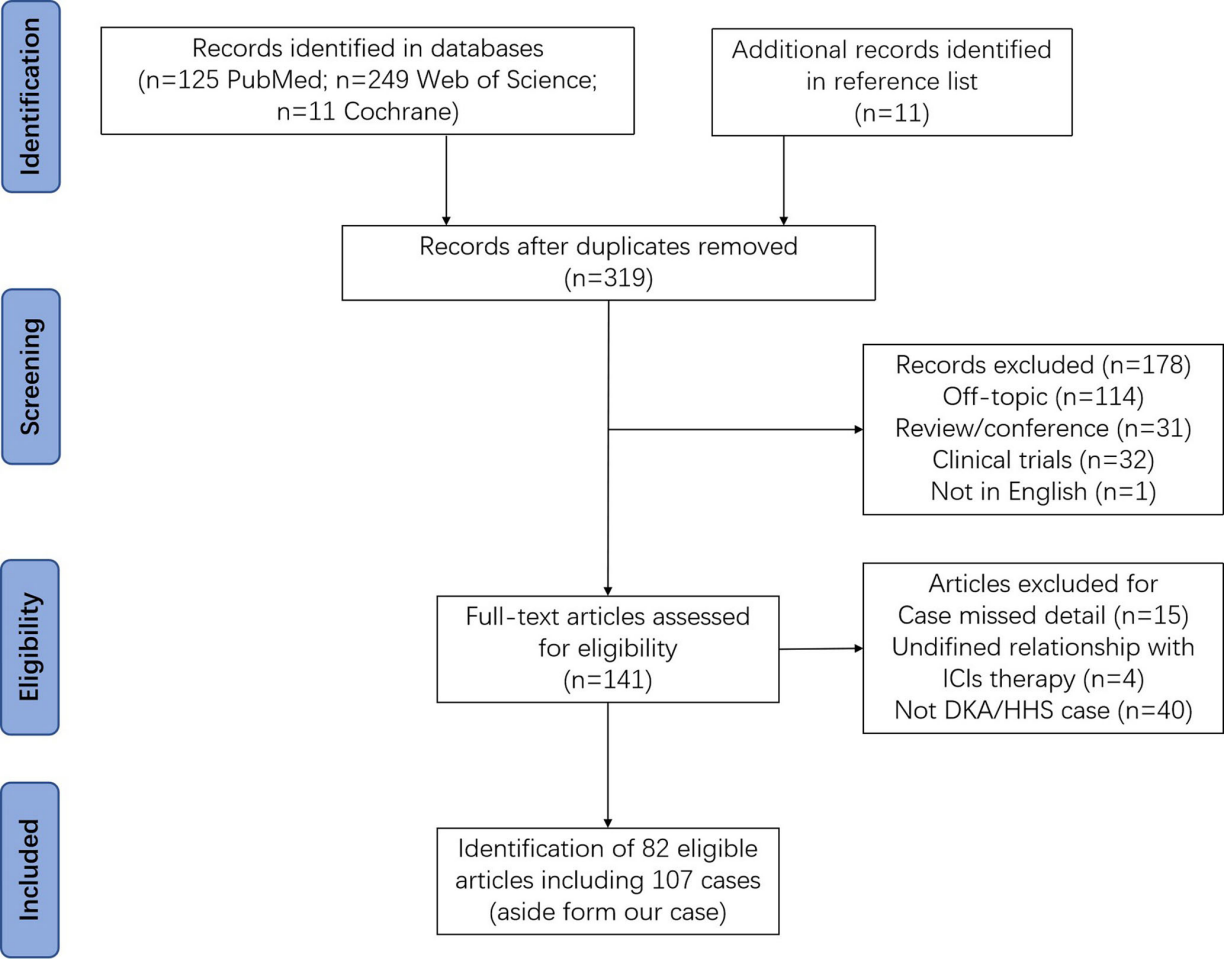
Flow diagram for the systematic review.

A total of 106 ICIs-DM cases were identified from 82 papers. The whole screen process was shown in the flow diagram in **Figure 1**. All detailed data of DKA are provided in **Supplementary Material S Table 1**. Amongst these 106 patients, 97 (91.5%) patients had DKA alone, while 9 (8.5%) patients had combined DKA-HHS (case 1 (7), case 2 (10), case 3 (11), case 4 (12), case 5 (13), case 6 (14), case 7 (15), case 8 (16)and case 9 (17) in **Supplementary Material S Table 3**). The detailed data have been presented in **Supplementary Material S Table 3**. Notably, the use of PD-1 inhibitors was reported in most patients. Most of the patients were male, and lung and melanoma were the two main treated cancer types in both groups. Acute renal failure and pancreatic exocrine dysfunction were developed in 87.5% (7/8) and 75% (3/4) cases, respectively. Two groups had similar risks for pancreatic exocrine dysfunction and concurrent infection.

### The main clinical characteristics of ICIs- induced combined DKA-HHS

The main clinical characteristics of ICIs- induced combined DKA-HHS were summarized in **Table 1**. Except for one patient without available data, all others had ICIs-DM with low levels of C-peptide or pH. The effective serum osmolarity of nine patients was further analyzed. All patients were above 320 mOsmol/kg H_2_O, 3 of which showed even higher than 340 mOsmol/kg H_2_O, 4 (cases 4, 5, 6, and 7 in **Supplementary Material S Table 3**) of which did not mention our research group calculated that according to the formula: 2*[measured serum Na+ (mEq/L)] + glucose (mg/dl)/18 referenced from ref (8). The glycemia was lower in the DKA group than in the combined DKA-HHS (686.7 ± 252.6 vs 1020 ± 102.5 mg/dL, p=0.0003). Patients with combined DKA-HHS were more likely to experience acute kidney than DKA ones (87.5% vs 28.6%, p=0.002), and Patients with combined DKA-HHS were more likely to have received chemotherapy prior to immunotherapy than patients with isolated DKA (66.7% vs 31.6%, p=0.034). Patients with combined DKA-HHS had significantly higher HbA1C levels than those without (9.2% vs 7.8%, p=0.027). The HbA1c results from long-term blood glucose control and is used as an indicator in clinical research (18), so we suspect these patients may have had impaired glucose tolerance before, but they have not been found.

**Table 1:** The clinical characteristics of combined DKA-HHS and DKA secondary to ICIs therapy.

	combined DKA-HHS (n=9)	DKA(n=98)	Estimated difference/OR, HHS vs. DKA (95% Cl)	*p* value
Age, years median (IQR)	69 (64-73)	62 (55-70)	7 (-2 to 12)	0.135
Sex, Male, n (%)	7/9 (77.8%)	59/98 (60.2%)	2.314(0.46 to 11.41)	0.477
History of DM, n (%)	3/9 (33.3%)	12/98 (12.2%)	3.58 (0.88 to 14.05)	0.112
History of immunological agents, n (%)	0/9 (0%)	13/98 (13.3%)	0.00 (0.00 to 3.07)	0.596
History of chemotherapy, n (%)	6/9 (66.7%)	31/98 (31.6%)	4.32 (1.34 to 16.34)	0.034
History of steroids usage, n (%)	2/9 (22.2%)	8/98 (8.2%)	3.21 (0.59 to 15.96)	0.198
Cancer type, n (%)				0.066
Melanoma	1/9 (11.1%)	35/98 (35.7%)	0.23 (0.02 to 1.63)	0.267
Lung	4/9(44.4%)	36/98 (36.7%)	1.38 (0.40 to 5.03)	0.725
Others	4/9 (44.4%)	27/98 (27.6%)	2.10 (0.60 to 7.80)	0.279
ICIs, n (%)				0.767
PD-1 monotherapy	6/9 (66.7%)	73/98 (74.5%)	0.68 (0.18 to 2.66)	0.694
PD-L1 monotherapy	2/9 (22.2%)	11/98 (11.2%)	2.26 (0.43 to 10.13)	0.300
CTLA-4 monotherapy	0/9 (0%)	1/98 (1.0%)	0.00 (0.00 to 98.00)	1
Combination therapy	1/9 (11.1%)	13/98 (13.3%)	0.82 (0.07 to 5.16)	1
Cycles to onset of ICI-DM	6 (9, 2.5-11.5)	3 (76, 2-8)	-3 (-5 to 1)	0.277
meadian (n, IQR)				
Glucose, mg/dl (mean ± SEM)	1020 ± 102.5	686.7 ± 252.6	333.7 ± 89.6 (156.1 to 511.3)	0.0003
G3	0/9(0%)	27.5% (27/98)	0.00 (0.00 to 1.13)	0.108
G4	9/9(100%)	72.5% (71/98)	0.00 (0.00 to 1.13)	0.108
HbA1c (%), meadian (n, IQR)	9.2 (9, 7.8-10.5)	7.8 (89, 7.1-8.7)	-1.4 (-2.40 to -0.20)	0.027
FDM, n (%)	1/9 (11.1%)	23/98 (23.5%)	0.41 (0.04 to 3.05)	0.680
Low serum C-peptide, n (%)	6/7 (85.7%)	66/73 (90.4%)	0.64 (0.07 to 8.26)	0.536
Autoantibody positivity, n (%)				1
Anti-GAD	3/9 (33.3%)	36/91 (39.6%)	1.05 (0.28 to 3.98)	1
Anti-IA2	0/3 (0%)	6/59 (10.2%)	0.90 (0.82 to 0.98)	1
Anti-ZnT8	0/2 (0%)	1/28 (3.6%)	0.96 (0.90 to 1.04)	1
Anti-ICA	0/3 (0%)	6/43 (14.0%)	0.89 (0.71 to 1.12)	1
Anti-insulin	0/2 (0%)	1/9 (11.1%)	0.86 (0.76 to 0.97)	1
HLA genes				0.712
risk factor, n (%)	3/3 (100%)	24/37 (64.9%)	0.64 (0.50 to 0.82)	0.284
protective factor, n (%)	0/3 (0%)	3/37 (8.1%)	0.92 (0.83 to 1.01)	1
both, n (%)	0/3 (0%)	1/37 (2.7%)	0.97 (0.92 to 1.03)	1
Complications				
acute kidney injury, n (%)	7/8 (87.5%)	20/70 (28.6%)	17.5 (2.69 to 200.10)	0.002
suspected infection, n (%)	1/4 (25%)	12/27 (44.4%)	0.36 (0.03 to 2.8)	0.606
elevated pancreatic exocrine gland lesion, n (%)	3/4 (75%)	17/39 (43.6%)	3.88 (0.37 to 40.71)	0.323

GAD, glutamic acid decarboxylase; IA_2_,islet antigen 2; ZnT8, zinc transporter 8; ICA, insulin cell antibodies; IAA, insulin autoantibodies; HLA, human leukocyte antigen.

G3^*^ represents patients with glucose levels between 13.9-27.8 mmol/L, and G4^*^ includes patients with glucose levels above 27.8 mmol/L, both of which were based on Brahmer JR, Lacchetti C, Schneider BJ, et al. National Comprehensive Cancer Network. Management of Immune-Related Adverse Events in Patients Treated with Immune Checkpoint Inhibitor Therapy: American Society of Clinical Oncology Clinical Practice Guideline. J Clin Oncol 2018; 36: 1714-1768. Acute renal failure*: creatinine or blood urea nitrogen was higher than the normal upper limit after ICIs therapy. Elevated exocrine dysfunction*: lipase or amylase was higher than the normal upper limit after ICIs therapy.

### Subgroup analysis of T1DM-related autoantibodies and HLA genotypes

**Figure 2:**
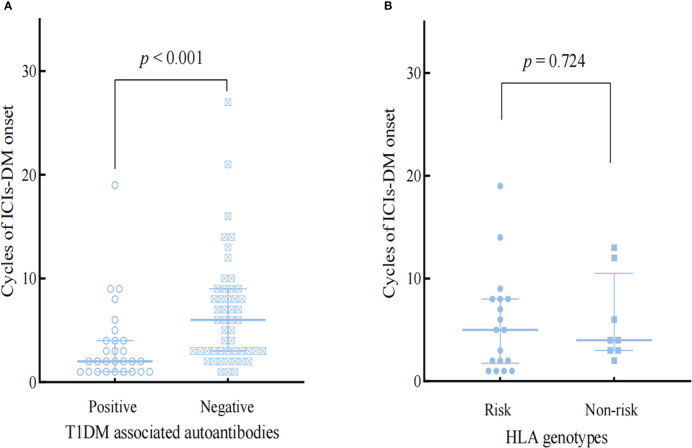
Comparison the influences of T1DM-associated autoantibodies **(A)** and HLA genotypes **(B)** on the onset of ICIs-DM.

**Figure 3:**
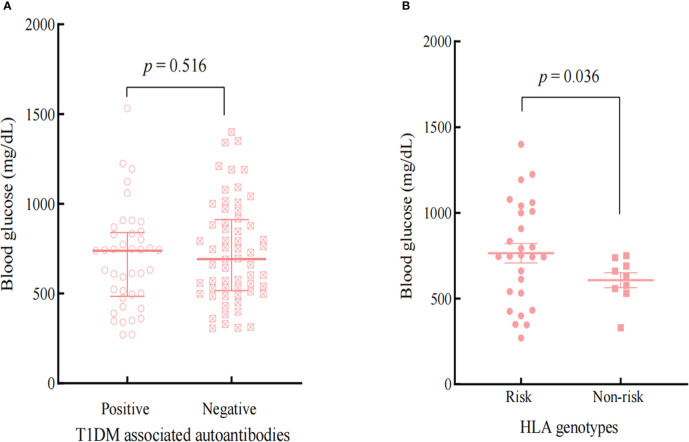
Comparison the effects of T1DM-associated autoantibodies **(A)** and HLA genotypes **(B)** on the BG at the onset of ICIs-DM.

T1DM -associated antibodies and genes within the HLA region are known to predispose a significant risk of developing Type 1 diabetes (19). Based on haplotypes DRB1*0401-DQB1*0302 (HLA-DR4-DQ8) and DRB1*0301-DQB1*0201 (HLA-DR3-DQ2) confer the most significant susceptibility for T1DM (19)and Kyle A et al.’s comprehensive analysis results on genes of immunosuppressant induced diabetes (20), we selected patients with HLA-DR4-DQ8 or HLA-DR3-DQ2 as risk groupings of HLA alleles. **Figure 2** and **Figure 3** showed the influences of T1DM-related autoantibodies and HLA risk factors on the presentation of ICIs-DM. We found that patients with positive autoantibody assumed significantly more acute onset of hyperglycemia complication as DKA or combined DKA-HHS than negative ones (2 vs 6 cycles, p<0.001) in **Figure 2**. Moreover, patients with the HLA risk factor genotype presented significantly higher serum glucose than non-risk ones (764.4 ± 297.0 vs 607.4 ± 129.5 mg/dL, p=0.036) shown in **Figure 3**. Neither autoantibody nor HLA genotype could affect the Hb1Ac level. Further investigation of T1DM-related autoantibody and HLA genotype tests showed anti-glutamic acid decarboxylase (GAD) antibody positivity and risk factor of DR4-DQ8 genotype as the predominance. Due to the limited sample size, we could not draw and rule out conclusions in ICIs - induced combined DKA-HHS groups. However, we found that time from initiation of immunotherapy to a diagnosis of combined DKA-HHS occurred more quickly in GAD antibody positivity (case 3, case 7, case 8 in **Supplementary Material S Table 3**) than negative (the other six cases). Hopefully, more focus and data on this subgroup will explore ICIs - induced combined DKA-HHS-related autoantibodies and HLA genotypes in the future.

## Discussion

No systematic analysis has been conducted on the clinical characteristics of combined DKA-HHS patients associated with ICIs. Based on existing data, clinicians may not pay sufficient attention to ICIs- induced combined DKA-HHS in that DKA remains the most common hyperglycemic emergency after immune checkpoint inhibitors, and combined DKA-HHS was a rare, mixed presentation with a combination of acidosis and/or ketonuria. Our results highlight the importance of identifying patients with combined DKA-HHS.

### The reason why the diagnosis of ICIs - induced combined DKA-HHS was neglected

In order to provide a better understanding of the characteristics of ICIs - induced combined DKA-HHS, we compare the clinical diagnosis, clinical manifestations, complications, and treatment. Based on previous reports, a subtype of type 1 diabetes was presented with ICIs- induced combined DKA-HHS according to the clinical course: rapidly increasing blood glucose over a short period of time, rapid pancreatic beta-cell destruction with low serum C-peptide and type 1 diabetes-associated antibody having been reported. From a clinical perspective, the most obvious feature in two groups exhibited hyperglycemic symptoms, such as dry mouth, polydipsia, and polyuria, and the period from the occurrence of hyperglycemic symptoms to the diagnosis of diabetic ketoacidosis or ketosis was approximately two weeks. Moreover, endogenous insulin secretion is depleted within three weeks of the clinical onset of ICIs-DM. Patients with ICIs- induced combined DKA-HHS exhibited unpredictable onset, occurring not only as early as a few weeks after initiation of immunotherapy but also up to a year since the discontinuation of treatment, ranging from 2 to 21 cycles, which still showed similarity. Similar performances were found between the two groups, which may be the main reason why the doctor was little attention and neglected the diagnosis.

We furthermore compared the clinical characteristics of the two groups regarding age, glucose, comorbidities, infection, acute kidney injury, T1DM-related autoantibody, as well as genetic factors. Apart from blood glucose, acute kidney, and previous exposure to chemotherapy, none of the biochemical parameters and clinical characteristics at presentation was significantly different between the DKA and combined DKA-HHS groups. It is worth noting that the majority of these patients had primary thyroid dysfunction (hypothyroidism or thyroiditis) in the DKA group, which is the most common cooccurrence with type 1 diabetes related to ICIs (21, 22). We recommend that blood glucose be frequently monitored for this population in that thyroid dysfunction is associated with T1DM and T2DM (23–25).

### Pathogenesis and predisposing factors of ICIs - induced combined DKA-HHS

Although the relationship between diabetes and immune checkpoint inhibitors has been widely studied, the exact pathophysiological mechanism of ICIs -DM is still not wholly unknown. There is growing evidence supporting such findings (26, 27), and in this autoimmune process, the PD-1/PD-L1 pathway is the most prominent for activating autoreactive T cells (28–30). As pancreatic β cells also express PD-1 ligand (PD-L1) but lack CTLA-4 receptors, the immune response stimulated by the ICIs monoclonal antibodies binding to PD-1/PD-L1 could lead to increased infiltration and destruction of pancreatic beta cells (31). This explained why most cases of ICIs-DM were caused by PD-1 monotherapy and needed permanent insulin therapy even after crisis relief.

We implied that ICIs- induced combined DKA-HHS could be caused by multiple physiological and pathological mechanisms based on the following reasoning: First, type 1 diabetes results from a deficiency of circulating insulin and increased levels of the counter-regulatory hormones catecholamines, glucagon, cortisol, and growth hormone. Immune checkpoint inhibitors destroy the pancreatic β-cells, which leads to loss of insulin secretion and absolute insulin deficiency. Second, infection, T1DM-related autoantibodies, and genetic factors have been identified as important increased risks in the pathogenesis of ICIs-DM (2, 28, 32–39), although it does not completely distinguish who will develop ICIs- induced combined DKA-HHS. In addition, the results of our systematic review confirmed that acute renal injury and chemotherapy history were significantly higher occurrence rates with the ICIs- induced combined DKA-HHS. The chemotherapeutic drugs they used included platinum, pemetrexed, cyclophosphamide, and paclitaxel, all of which can cause kidney damage. A large cohort study confirmed that acute kidney injury was associated with a 5.5-fold increase in mortality in HHS cases (40). It has been reported that cisplatin can cause HHS (33, 34), but the mechanism has not been described. Finally, we found more risk factors in the group with ICIs- induced combined DKA-HHS. We thus hypothesized that multiple factors might act additively or synergistically to contribute to severe hyperglycemia.

### Treatment of ICIs- induced combined DKA-HHS

There are limited data to guide the management of ICIs- induced combined DKA-HHS, so management strategies are also not clear for individuals with ICIs- induced combined DKA-HHS. Treatment of this case and other cases with ICIs- induced combined DKA-HHS in our literature were mainly based on ICIs-DM (41), including holding ICIs until the patient is clinically stable and glycemic control is reached, vigorous intravenous rehydration, electrolyte replacement, and insulin therapy. Concerning the management of patients with a mixed presentation, as indicated in the current guidelines (42) and suggestion of ref (40) for combined DKA-HHS patients, it is similar to those of DKA but with greater intravenous fluid administration and close monitoring of circulation to ensure adequate hydration. Besides, ketone production and acidosis begin to resolve with insulin treatment, but insulin drives potassium back into the cell, and the patient can become severely hypokalemic. Therefore, we recommend that providers taking care of patients with ICIs- induced combined DKA-HHS hold immunosuppressive therapy and give insulin treatment. Moreover, clinicians should pay enough attention to frequently monitor sodium and potassium and administer adequate intravenous fluid.

## Conclusions

In conclusion, we highlight combined DKA-HHS as an unusual presentation and complication of ICIs-T1DM.A mixed presentation with features of DKA and HHS is a frequent yet underrecognized and underreported emergency in ICIs-DM. Identifying hyperosmolarity is essential, as its treatment requires more-aggressive fluid administration than treatment of ICI-DKA, and improper management may increase the risk of complications. Our data show that increased exposure to more risk factors accounts for the increased incidence of ICIs-induced combined DKA-HHS. It is the first retrospective, observational case series summarizing the clinical characteristics of combined DKA-HHS patients associated with ICIs. We hope that clinicians gain more excellent experience in the side effects of still relatively immune checkpoint inhibitors and that new cases of ICIs-induced combined DKA-HHS will be detected earlier.

## Author contributions

WZ and JC took part in all data analysis, writing and editing of the manuscript. JB and ND involved the screening and identification of all case reports. XC reviewed and edited the manuscript especially in language spelling. YJ was responsible for the management of our patient case. ZW mainly contributed to the critical revision of the manuscript and supervision of all edition processes. All authors contributed to the article and approved the submitted version.
