# Supplementary material for: Combined diabetic ketoacidosis and hyperosmolar hyperglycemic state in type 1 diabetes mellitus induced by immune checkpoint inhibitors: Underrecognized and underreported emergency in ICIs-DM
Source: Front Endocrinol (Lausanne). 2023 Jan 4;13:1084441. doi: 10.3389/fendo.2022.1084441 (PMC9846077; doi:10.3389/fendo.2022.1084441)
Supplement: Supplementary file 1 [file Table_1.docx]

**S Table 1|** Search strategies on pubmed, Web of Science and Cochrane

Search strategy on Pubmed:

((ipilimumab[Text Word] OR tremelimumab[Text Word] OR ticilimumab[Text Word] OR pembrolizumab[Text Word] OR nivolumab[Text Word] OR atezolizumab[Text Word] OR avelumab[Text Word] OR durvalumab[Text Word] OR camrelizumab[Text Word] OR toripalimab[Text Word] OR sintilimab[Text Word] OR tislelizumab[Text Word]) OR ((immune checkpoint inhibitor[Text Word] OR immune checkpoint blockade[Text Word]) OR ((anti-programmed cell death receptor 1[Text Word] OR anti-PD-1[Text Word] OR programmed cell death receptor 1 inhibitor) OR (anti-programmed cell death receptor ligand 1[Text Word] OR anti-PD-L1[Text Word] OR programmed cell death receptor ligand 1 inhibitor[Text Word] OR PD-L1 inhibitor[Text Word]) OR (anti-cytotoxic T-lymphocyte antigen 4[Text Word] OR anti-CTLA-4[Text Word] OR cytotoxic T-lymphocyte antigen 4 inhibitor[Text Word] OR anti-CTLA-4 inhibitor[Text Word])))) AND (diabet*[MeSH Terms] OR hyperglycemia[MeSH Terms] OR insulin*[MeSH Terms]) AND English[Language] AND case[Text Word]

Search strategy on Web of science:

(TS=(ipilimumab OR tremelimumab OR ticilimumab OR pembrolizumab OR nivolumab OR atezolizumab OR avelumab OR durvalumab OR camrelizumab OR toripalimab OR sintilimab OR tislelizumab) OR TS=(immune checkpoint inhibitor OR immune checkpoint blockade) OR TS=((anti-programmed cell death receptor 1 OR anti-PD-1 OR programmed cell death receptor 1 inhibitor OR PD-1inhibitor) OR (anti-programmed cell death receptor ligand 1 OR PD-L1 OR programmed cell death receptor ligand 1 inhibitor OR PD-L1 inhibitor) OR (anti-cytotoxic T-lymphocyte antigen 4 OR anti-CTLA-4 OR cytotoxic T-lymphocyte antigen 4 inhibitor OR CTLA-4 inhibitor))) AND TS=(diabet* OR hyperglycemia OR insulin*) AND LA=(English) AND ALL=(case)

Search strategy on Cochrane:

| **Set** | **Search** |
| --- | --- |
| #1 | (ipilimumab):ti,ab,kw |
| #2 | (tremelimumab):ti,ab,kw |
| #3 | (ticilimumab):ti,ab,kw |
| #4 | (pembrolizumab):ti,ab,kw |
| #5 | (nivolumab):ti,ab,kw |
| #6 | (atezolizumab):ti,ab,kw |
| #7 | (avelumab):ti,ab,kw |
| #8 | (durvalumab):ti,ab,kw |
| #9 | (camrelizumab):ti,ab,kw |
| #10 | (toripalimab):ti,ab,kw |
| #11 | (sintilimab):ti,ab,kw |
| #12 | (tislelizumab):ti,ab,kw |
| #13 | #1 OR #2 OR #3 OR #4 OR #5 OR #6 OR #7 OR #8 OR #9 OR #10 OR #11 OR #12 |
| #14 | (immune checkpoint inhibitor):ti,ab,kw |
| #15 | (immune checkpoint blockade):ti,ab,kw |
| #16 | #14 OR #15 |
| #17 | (anti-programmed cell death receptor 1):ti,ab,kw |
| #18 | (anti-PD-1):ti,ab,kw |
| #19 | (programmed cell death receptor 1 inhibitor):ti,ab,kw |
| #20 | (PD-1 inhibitor):ti,ab,kw |
| #21 | #17 OR #18 OR #19 OR #20 |
| #22 | (anti-programmed cell death receptor ligand 1):ti,ab,kw |
| #23 | (anti-PD-L1):ti,ab,kw |
| #24 | (programmed cell death receptor ligand 1 inhibitor):ti,ab,kw |
| #25 | (PD-L1 inhibitor):ti,ab,kw |
| #26 | #22 OR #23 OR #24 OR #25 |
| #27 | (anti-cytotoxic T-lymphocyte antigen 4):ti,ab,kw |
| #28 | (anti-CTLA-4):ti,ab,kw |
| #29 | (cytotoxic T-lymphocyte antigen 4 inhibitor):ti,ab,kw |
| #30 | (CTLA-4 inhibitor):ti,ab,kw |
| #31 | #27 OR #28 OR #29 OR #30 |
| #32 | #21 OR #26OR #31 |
| #33 | #13 OR #16 OR #32 |
| #34 | (diabet*):ti,ab,kw |
| #35 | (hyperglycemia):ti,ab,kw |
| #36 | (insulin*):ti,ab,kw |
| #37 | #34 OR #35 OR #36 |
| #38 | (case) |
| #39 | #33 AND #37 AND #38 |
