# Supplementary material for: Combined diabetic ketoacidosis and hyperosmolar hyperglycemic state in type 1 diabetes mellitus induced by immune checkpoint inhibitors: Underrecognized and underreported emergency in ICIs-DM
Source: Front Endocrinol (Lausanne). 2023 Jan 4;13:1084441. doi: 10.3389/fendo.2022.1084441 (PMC9846077; doi:10.3389/fendo.2022.1084441)
Supplement: Supplementary file 2 [file Table_2.docx]

**S Table 2|** Detailed information of ICIs-DKA cases

| Ref. | ICIs^a^ | Sex | Age | History of DM^b^ | History of immunological agent^c^ | History of chemotheray | Tumor type^d^ |  | DM type^e^ | Other irAE^f^ | HLA genes^g^ | Acute renal injury | History of infection | Pancreatic enzyme elevation^h^ | Glycose (mg/dl) | HbA1c (%) | C-peptide^i^ | History of steroids | Autoantibody^j^j | cysles |
| --- | --- | --- | --- | --- | --- | --- | --- | --- | --- | --- | --- | --- | --- | --- | --- | --- | --- | --- | --- | --- |
| 1 | N | F | 68 | - | - | - | M |  | T1DM | T+A | NA | - | NA | - | 310 | 8.2 | L | Y | - | 27 |
| 2 | P | F | 51 | - | - | Y | O |  | T1DM | O | NA | - | NA | NA | 1123 | 8.3 | L | - | GAD | 2 |
| 3 | P | F | 73 | - | - | - | O |  | T1DM | A | R | - | NA | - | 540 | 7.1 | L | - | - | 2 |
| 4 | N | M | 73 | - | INF | - | M |  | FT1DM | G | NA | Y | NA | NA | 500 | 8.8 | U | NA | GAD IA2 ICA | 3 |
| 5 | A | M | 57 | - | - | Y | U |  | T1DM | - | R | - | NA | NA | 432 | 7.5 | L | - | NA | 5 |
| 6 | N | M | 77 | - | - | - | O |  | IDDM | - | NA | - | Y | NA | 801 | 8.3 | NA | - |  | 15 |
| 7 | P+I | M | 70 | - | - | - | L |  | DM | A | NA | Y | - | - | 794 | 6.5 | L | - |  | 3 |
| 8 | N | M | 68 | - | - | - | M |  | T1DM | - | NA | Y | Y | NA | 643 | NA | L | NA | - | 2 |
| 9 | P | M | 12 | F | - | - | O |  | T1DM | O | R | - | - | NA | 347 | 8.9 | L | NA | IA2 | 5 |
| 10 | N+I | F | 60 | - | - | - | R |  | T1DM | - | - | - | NA | - | 330 | 6.5 | L | Y |  | 3 |
| 11 | N | M | 59 | - | - | - | O |  | T1DM | - | - | - | - | NA | 690 | 10.6 | L | NA | - | 12 |
| 12 | S | M | 59 | - | - | Y | L |  | T1DM | O | NA | - | NA | NA | 450 | NA | L | NA | - | 5 |
| 13 | N | F | 66 | - | - | - | M |  | FT1DM | - | - | - | - | - | 531 | 7.3 | L | NA | - | 6 |
| 14 | N+I | M | 42 | - | - | - | M |  | IDDM | C | NA | Y | - | NA | 728 | 6.5 | NA | NA | - | 3 |
| 15 | N | F | 63 | - | - | - | M |  | DM | - | NA | - | NA | A | 661 | 8.9 | L | NA | - | 8 |
| 16 | P | F | 67 | F | - | Y | O |  | T1DM | - | NA | - | NA | - | 475 | 8.7 | NA | Y | GAD | 9 |
| 17 | N | F | 34 | - | - | Y | L |  | DM | T | - | - | NA | NA | 739 | 7.1 | L | NA | GAD IA2 ICA ZnT8 | 2 |
| 18 | N | M | 31 | - | - | - | L |  | FT1DM | - | R | - | NA | NA | 743 | 6.4 | L | NA | GAD | 1 |
| 19 | A | F | 74 | - | - | Y | O |  | DM | - | NA | Y | Y | NA | 845 | 7.5 | L | NA | - | 14 |
| 20 | A | M | 64 | T2DM | INF | - | R |  | T1DM | - | NA | - | NA | NA | 609 | 8.3 | NA | Y | GAD | 8 |
| 21 | N | M | 55 | - | - | Y | L |  | T1DM | - | NA | - | NA | NA | 498.6 | 8.2 | U | NA | - | 9 |
| 22 | N | F | 73 | - | - | - | L |  | FT1DM | - | R | Y | Y | NA | 1000 | 7.2 | L | NA | - | 2 |
| 23 | P | M | 55 | - | ICIs | - | M |  | T1DM | H | NA | - | NA | NA | 756 | NA | NA | Y | - | 9 |
| 24 | A | F | 70 | - | - | - | L |  | DM | - | NA | - | NA | NA | 1015 |  | N | NA | - | 3 |
| 25 | N | M | 31 | - | - | - | L |  | FT1DM | - | R | - | NA | NA | 743 | 6.4 | U | NA | GAD | 1 |
| 26 | A | M | 82 | - | - | - | L |  | DM | - | R | Y | NA | NA | 1078 | 8.6 | L | NA | - | 8 |
| 27 | I | M | 64 | - | INF | - | M |  | FT1DM | H | - | - | NA | NA | 750 | 6.1 | L | Y | - | 3 |
| 28 | N | F | 74 | - | - | - | M |  | FT1DM | - | NA | - | - | A | 571 | 8 | NA | NA | - | 6 |
| 29 | N+I | M | 51 | - | - | - | R |  | FT1DM | O | NA | - | NA | L | 763 | 7.2 | U | NA | - | 2 |
| 30 | N+I | M | 60 | T2DM | - | - | M |  | T1DM | O | NA | - | NA | L | 828 | 7.6 | NA | NA | GAD IA2 ICA | 1 |
| 31 | N+I | F | 80 | - | - | - | M |  | T1DM | T | NA | - | NA | NA | 871 | NA | NA | NA | GAD ICA | 2 |
| 32 | A+4 | M | 50 | - | - | - | O |  | T1DM | - | NA | - | NA | - | 340.2 | 6.4 | L | NA | GAD | 2 |
| 33 | P | M | 60 | - | ICIs | - | M |  | T1DM | T | NA | - | NA | - | 486 | 7.1 | L | NA | - | 2 |
| 34 | S | M | 42 | - | - | - | O |  | T1DM | O | NA | - | NA | NA | 306 | NA | U | NA | - | 10 |
| 35 | N | F | 63 | - | - | - | M |  | FT1DM | - | R | - | NA | NA | 661 | 8.6 | L | NA |  | 8 |
| 36 | P | M | 62 | - | - | - | L |  | T1DM | O | NA | Y | - | - | 1191 | 11 | L | NA | - | 3 |
| 37 | P | M | 53 | - | - | - | L |  | FT1DM | - | NA | Y | Y | L | 630 | 7.2 | U | NA | GAD | 2 |
| 38 | N | M | 42 | F | - | - | L |  | FT1DM | - | - | - | NA | - | 632 | 6 | L | - | GAD | 4 |
| 39 | N+I | F | 49 | - | - | Y | L |  | T1DM | - | NA | - | NA | - | 384 | 6.6 | L | - | - | 2 |
| 40 | P | F | 47 | - | - | Y | O |  | T1DM | - | NA | - | - | NA | 523 | 6.4 | L | - | GAD | 1 |
| 41 | A | M | 62 | - | ICIs | Y | L |  | FT1DM | - | R | Y | Y | NA | 1041 | 6.9 | L | - | - | 1 |
| 42 | N | M | 83 | - | - | - | L |  | T1DM | T | R | - | Y | NA | 426 | 7.4 | L | - | GAD | 6 |
| 43 | A | F | 63 | - | - | - | O |  | T1DM | T | R | - | NA | NA | 801 | 7.8 | L | - | GAD | 9 |
| 44 | P | M | 76 | - | - | - | L |  | DM | A | NA | - | NA | NA | 493 | 10.4 | L | - | - | 3 |
| 45 | P | F | 78 | T2DM | - | - | M |  | DM | - | NA | - | NA | NA | 494 | 11.4 | L | - | GAD | 1 |
| 46 | P | F | 65 | - | - | - | O |  | DM | - | NA | - | NA | NA | 511 | 5.8 | L | - | - | 7 |
| 47 | P | F | 68 | - | - | Y | L |  | T1DM | T | NA | Y | - | - | 882 | 7 | L | - | - | 6 |
| 48 | N | F | 87 | - | - | - | M |  | T1DM | - | NA | Y | Y | - | 768.6 | 8.5 | L | - |  | 3 |
| 49 | P | F | 77 | - | - | Y | O |  | DM | - | NA | - | NA | NA | 747 | 8.8 | NA | - | - | 16 |
| 50 | N | M | 67 | T2DM | - | Y | L |  | DM | - | NA | - | Y | NA | 514.8 | 7.6 | L | - | GAD | 1 |
| 51 | P | F | 85 | - | - | - | O |  | DM | - | NA | - | NA | NA | 555 | 6.8 | NA | - | - | NA |
| 52 | D | F | 49 | - | - | - | L |  | T1DM | - | NA | - | NA | NA | 361.8 | 7.8 | L | - | GAD | NA |
| 53 | N | F | 51 | - | - | Y | L |  | FDM | - | R | - | NA | L | 1224 | 8 | L | - | GAD | NA |
| 54 | N | F | 61 | - | - | Y | L |  | FDM | - | R | - | NA | NA | 1400 | 9.3 | L | - | - | NA |
| 55 | S | M | 56 | - | - | - | O |  | DM | - | R | - | - | NA | 399.6 | 7.8 | N | - | - | 8 |
| 56 | P | M | 70 | T2DM | - | - | M |  | T1DM | - | NA | - | NA | NA | 390 | 11.9 | U | - | GAD IAA | NA |
| 57 | N | M | 67 | - | ICIs | Y | L |  | T1DM | - | NA | - | NA | NA | 971 | 9.2 | U | - | - | NA |
| 58 | N | M | 49 | - | - | Y | L |  | T1DM | O | NA | - | NA | NA | 773 | 8.9 | U | - | GAD | NA |
| 59 | N | M | 27 | - | ICIs | - | M |  | T1DM | A | NA | - | NA | NA | 314 | 6.2 | U | - | - | NA |
| 60 | N | F | 74 | - | - | Y | L |  | T1DM | T | NA | - | NA | NA | 896 | 9.3 | U | - | - | NA |
| 61 | N | F | 66 | - | - | - | O |  | AT1DM | T | R | NA | NA | NA | 751.86 | 9.4 | L | - | GAD ICA | 3 |
| 62 | P | F | 61 | - | - | Y | L |  | FT1DM | T | NA | - | - | A | 572 | 8.4 | L | - | - | 8 |
| 63 | P | M | 67 | - | - | Y | L |  | ADM | - | NA | NA | NA | - | 485.64 | 8 | L | - | - | 3 |
| 64 | P | M | 61 | - | - | - | L |  | ADM | T | R | Y | Y | L | 1194 | NA | L | - | GAD | NA |
| 65 | P | M | 52 | - | - | - | M |  | T1DM | T | NA | NA | NA | A | 694.8 | 8.3 | L | - | - | 7 |
| 66 | N+I | F | 65 | - | D+T | - | R |  | T1DM | T | NA | NA | NA | - | 603 | 8.5 | L | - | - | 4 |
| 67 | N | M | 63 | - | - | Y | N |  | T1DM | T | NA | NA | NA | NA | 592 | 7.2 | NA | - | GAD | 4 |
| 68 | P | M | 82 | - | - | - | O |  | T1DM | P | NA | Y | NA | B | 1350 | NA | L | - | - | 1 |
| 69 | P | F | 81 | - | - | - | M |  | IDDM | - | NA | Y | - | L | 858.6 | 8.9 | NA | - | - | 8 |
| 70 | P | M | 75 | - | - | Y | U |  | FT1DM | - | NA | NA | NA | - | 1092 | 6.7 | U | - | - | 3 |
| 71 | N | M | 62 | - | - | - | M |  | T1DM | - | R | Y | NA | NA | 834 | NA | NA | - | GAD | 1 |
| 72 | P | M | 62 | - | - | Y | L |  | T1DM | - | NA | Y | Y | - | 1191 | 11 | L | - | - | 3 |
| 73 | D | M | 55 | F | - | - | O |  | T1DM | T | NA | NA | NA | NA | 417 | 8.4 | L | - | GAD IA2 | 1 |
| 74 | N | M | 49 | - | - | - | R |  | IDDM | - | NA | NA | NA | - | 799.2 | 10.9 | NA | - | NA | 21 |
| 75 | P+I | M | 59 | - | NA | NA | M |  | ADM | T | - | NA | NA | - | 660.6 | 6.8 | L | NA | - | NA |
| 76 | N+I | M | 61 | - | NA | NA | M |  | ADM | C+P | NA | NA | NA | B | 795.6 | 7.7 | L | NA | - | NA |
| 77 | N+I | M | 64 | - | NA | NA | M |  | ADM | - | NA | NA | NA | B | 360 | 9.8 | L | NA | - | NA |
| 78 | P | M | 55 | - | NA | NA | M |  | ADM | P+h | P | NA | NA | B | 612 | 13.1 | L | NA | GAD | NA |
| 79 | P | M | 70 | N | RA/C | - | M |  | T1DM | P | NA | NA | NA | NA | 900 | 8.9 | NA | NA | GAD | NA |
| 80 | P | M | 61 | N | - | - | M |  | T1DM | - | R | NA | NA | NA | 270 | 7.7 | L | NA | GAD | NA |
| 81 | P | F | 53 | N | - | Y | M |  | T1DM | - | R | NA | NA | NA | 612 | 7.2 | L | NA | GAD | NA |
| 82 | P | M | 23 | N | - | - | M |  | T1DM | T | R | NA | NA | NA | 745.2 | 7.5 | L | NA | GAD ICA | NA |
| 83 | P | M | 61 | N | I | - | M |  | T1DM | - | B | NA | NA | NA | 271.8 | 8.6 | L | NA | GAD | NA |
| 84 | N | M | 50 | N | I | - | M |  | T1DM | - | R | NA | NA | NA | 747 | 6.3 | L | NA | GAD | NA |
| 85 | P | M | 65 | T2DM | - | - | M |  | T1DM | - | NA | NA | NA | NA | 432 | 7.4 | L | NA | - | 1 |
| 86 | N | M | 55 | - | - | Y | L |  | FDM | C | P | NA | NA | NA | 498.6 | 8.2 | L | - | - | 9 |
| 87 | D | M | 69 | - |  | Y | L |  | FDM | R | - | NA | NA | NA | 558 | 7.4 | L | - | - | 13 |
| 88 | P | M | 83 | - |  | - | M |  | FDM | HD | - | NA | NA | L | 576 | 8.5 | L | - | - | 4 |
| 89 | N+I | F | 65 | - |  |  | M |  | FDM | HE | P | NA | NA | L | 792 | 7.3 | L | - | - | 5 |
| 90 | N | F | 55 | - | I | - | M |  | ADM | AT | R | NA | NA | NA | 532 | 6.9 | L | Y | - | NA |
| 91 | N | F | 83 | - | - | - | L |  | ADM | NA | R | NA | NA | NA | 350 | 7.7 | L | NA | GAD | NA |
| 92 | P | M | 58 | T2DM | - | Y | L |  | ADM | NA | NA | NA | NA | NA | 749 | 9.7 | L | NA | GAD | 1 |
| 93 | P | F | 41 | - | I |  | M |  | T1DM | T | R | NA | NA | NA | 908.1 | 6.8 | L | NA | IA2 | 19 |
| 94 | P | M | 73 | - | - | Y | L |  | ADM | - | NA | Y | Y | NA | 917.64 | 7.6 | L | - | - | 10 |
| 95 | N | M | 70 | - | - | - | R |  | ADM | - | NA | - | - | L | 878 | 8.4 | L | - | - | 3 |
| 96 | A | F | 73 | - | - | Y | L |  | FT1DM | HD | NA | Y | - | - | 962 | 7.3 | U | - | - | 2 |
| 97 | N | F | 56 | - | - | Y | L |  | FT1DM | - | NA | Y | NA | - | 846 | 8.2 | U | - | GAD | 3 |
| 98 | P | F | 61 | F | - | Y | B |  | FT1DM | - | NA | NA | - | - | 602.64 | 8.4 | L | - | - | 7 |

*^a^: N: nivolumab, P: pembrolizumab, A: atezolizumab, I: ipilimumab, S: sintilimab, D: durvalumab, 4: 4-1BB, T: tremelimumab.*

*^b^: F: family history of DM.*

*^c^: INF: interferon, D: durvalumab, T: tremelimumab, RA: rheumatoid arthritis, C: Covid, I: ipilimumab.*

*^d^: M: malignant melanoma, O: others, L: lung cancer, U: urothelial carcinoma, R: renal cell cancer, B: breast cancer.*

*^e^: T1DM: type 1 diabetes mellitus, FT1DM: fulminant type 1 diabetes mellitus, ADM: autoimmune diabetes mellitus, IDDM: insulin-dependent diabetes mellitus, FDM: fulminant diabetes mellitus.*

*^f^: T: thyroiditis, A: adrecortical insufficiency, G: Graves, C: colitis, H: hypophysitis, P: pancreatitis, h: hepatitis, R: rash, AT: autoimmune thyroid disease, O: others, HD: Hashimoto’s disease, HE: hypereosinophilia.*

*^g^: R: risk factor, P: protective factor, B: both of risk and protective factors.*

*^h^: L: lipase, A: amylase, B: both of lipase and amylase.*

*^i^: L: low C-peptide level, U: undetectable C-peptide level, N: normal C-peptide level.*

*^j^: GAD: glutamic acid decarboxylase, ZnT8: zinc transporter 8; IA2: islet antigen 2; ICA: insulin cell antibody; IAA: insulin autoantibody.*

Reference

1. Sakaguchi C, Ashida K, Yano S, Ohe K, Wada N, Hasuzawa N, et al. A case of nivolumab-induced acute-onset type 1 diabetes mellitus in melanoma. *Curr Oncol* (2019) 26:e115-e118. doi: 10.3747/co.26.4130

2. Kedzior SK, Jacknin G, Hudler A, Mueller SW, Kiser TH. A Severe Case of Diabetic Ketoacidosis and New-Onset Type 1 Diabetes Mellitus Associated with Anti-Glutamic Acid Decarboxylase Antibodies Following Immunotherapy with Pembrolizumab. *Am J Case Rep* (2021) 22:e931702. doi: 10.12659/AJCR.931702

3. Clotman K, Janssens K, Specenier P, Weets I, Block C. Programmed cell death-1 inhibitor-induced type1 diabetes mellitus. *J Clin Endocrinol Metab* (2018) 103:3144-3154. doi: 10.1210/jc.2018-00728

4. Gauci ML, Laly P, Tiphaine VT, Baroudjian B, Gottlieb J, Madilessi N, et al. Autoimmune diabetes induced by PD-1 inhibitor-retrospective analysis and pathogenesis: a case report and literature review. *Cancer Immunol Immunother* (2017) 66:1399-1410. doi: 10.1007/s00262-017-2033-8

5. Hickmott L, Hugo DL, Turner H, Ahmed F, Protheroe A, Grossman A, et al. Anti-PD-L1 atezolimumab-induced autoimmune diabetes: a case report and review of the literature. *Target Oncol* (2017) 12:235-241. doi: 10.1007/s11523-017-0480-y

6. Delasos Lukas, Bazewicz C, Sliwinska A, Lia NL, Vredenburgh J. New onset diabetes with ketoacidosis following nivolumab immunotherapy: A case report and review of literature. *J Oncol Pharm Pract* (2021) 27:716-721. doi: 10.1177/1078155220943949

7. Porntharukchareon T, Tontivuthikul B, Sintawichai N, Srichomkwun P. Pembrolizumab and ipilimumab induced diabetic ketoacidosis and isolated adrenocorticotropic hormone deficiency: a case report. *J Med Case Rep* (2020) 14:171. doi: 10.1186/s13256-020-02502-w

8. Abdullah H, Elnair R, Khan UI, Omar M, Oscar LM. Rapid onset type-1 diabetes and diabetic ketoacidosis secondary to nivolumab immunotherapy: a review of existing literature. *BMJ Case Rep* (2019) 12:e229568. doi: 10.1136/bcr-2019-229568

9. Samoa RA, Lee HS, Kil SH, Roep BO. Anti-PD-1 Therapy-Associated Type 1 Diabetes in a Pediatric Patient with Relapsed Classical Hodgkin Lymphoma. *Diabetes Care* (2020) 43:2293-2295. doi: 10.2337/dc20-0740

10. Yaura K, Sakurai K, Niitsuma S, Sato R, Takahashi K, Arihara Z. Fulminant type 1 diabetes mellitus developed about half a year after discontinuation of immune checkpoint inhibitor combination therapy with nivolumab and ipilimumab: a case report. *Tohoku J Exp Med* (2021) 254:253-256. doi: 10.1620/tjem.254.253

11. Mae S, Kuriyama A, Tachibana H. Diabetic ketoacidosis as a delayed immune-related event after discontinuation of nivolumab. *J Emerg Med* (2021) 60:342-344. doi: 10.1016/j.jemermed.2020.09.023

12. Huang XF, Yang M, Wang L, Li LB, Zhong XW. Sintilimab induced diabetic ketoacidosis in a patient with small cell lung cancer: A case report and literature review. *Medicine (Baltimore)* (2021) 100:e25795. doi: 10.1097/MD.0000000000025795

13. Miyoshi Y, Ogawa O, Oyama Y. Nivolumab, an anti-programmed cell death-1 antibody, induces fulminant type 1 diabetes. *Tohoku J Exp Med* (2016) 239:155-8. doi: 10.1620/tjem.239.155

14. Changizzadeh PN, Mukkamalla S, Armenio VN. Combined checkpoint inhibitor therapy causing diabetic ketoacidosis in metastatic melanoma. *J Immunother Cancer* (2017) 5:97. doi: 10.1186/s40425-017-0303-9

15. Teramoto Y, Nakamura Y, Asami Y, Imamura T, Takahira S, Nemoto M, et al. Case of type 1 diabetes associated with less-dose nivolumab therapy in a melanoma patient. *J Dermatol* (2017) 44:605-606. doi: 10.1111/1346-8138.13486

16: Matthew SC, Gill D, Voorhies BN, Agarwal N, Ignacio GL. Case report: pembrolizumab-induced Type 1 diabetes in a patient with metastatic cholangiocarcinoma. *Immunotherapy* (2017) 9:797-804. doi: 10.2217/imt-2017-0042

17. Godwin JL, Jaggi S, Sirisena I, Sharda P, Rao AD, Mehra R, et al. Nivolumab-induced autoimmune diabetes mellitus presenting as diabetic ketoacidosis in a patient with metastatic lung cancer. *J Immunother Cancer* (2017) 5:40. doi: 10.1186/s40425-017-0245-2

18. Usui Y, Udagawa H, Matsumoto S, Imai K, Ohashi K, Ishibashi M, et al. Association of Serum Anti-GAD Antibody and HLA Haplotypes with Type 1 Diabetes Mellitus Triggered by Nivolumab in Patients with Non-Small Cell Lung Cancer. *J Thorac Oncol* (2017) 12:e41-e43. doi: 10.1016/j.jtho.2016.12.015

19. Clontz R, Dang DM, Hieger MA, Becker BA. Atezolizumab-induced Autoimmune Diabetes in a Patient with Metastatic Breast Cancer: A Case Report. *Clin Pract Cases Emerg Med* (2021) 5:190-193. doi: 10.5811/cpcem.2021.2.51508

20. Rahman W, Conley A, Silver KD. Atezolizumab-induced type 1 diabetes mellitus in a patient with metastatic renal cell carcinoma. BMJ Case Rep. 2020;13(7):e233842.

21. Marchand L, Paulus V, Fabien N, Perol M, Thivolet C, Vouillarmet J, et al. Nivolumab-induced acute diabetes mellitus and hypophysitis in a patient with advanced pulmonary pleomorphic carcinoma with a prolonged tumor response. *J Thorac Oncol* (2017) 12:e182-e184. doi: 10.1016/j.jtho.2017.07.021

22. Araujo M, Ligeiro D, Costa L, Marques F, Trindade H, Correia JM, et al. A case of fulminant type 1 diabetes following anti-PD1 immunotherapy in a genetically susceptible patient. *Immunotherapy* (2017) 9:531-535. doi: 10.2217/imt-2017-0020

23. Humayun MA, Poole R. A case of multiple immune toxicities from ipilimumab and pembrolizumab treatment. *Hormones (Athens)* (2016) 15:303-306. doi: 10.14310/horm.2002.1656

24. Patti, R., Malhotra, S., Sinha, A., Singh, P., Marcelin, M., Saxena, A. Atezolizumab-induced new onset diabetes millitus with ketoacidosis. *Am J Ther* (2018) 25, e565-e568. doi: 10.1097/MJT.0000000000000644

25. Usui Y, Udagawa H, Matsumoto S, Imai K, Ohashi K, Ishibashi M, et al. Association of serum anti-GAD antibody and HLA haplotypes with type 1 diabetes mellitus triggered by nivolumab in patients with non-small cell lung cancer. *J Thorac Oncol* (2017) 12:e41-e43. doi: 10.1016/j.jtho.2016.12.015

26. Rodríguez, D. V. G. P., Tous, R. M., Morales, P. C., Olmedo I.S., Brocca, M.A.M. Diabetes mellitus associated with immune checkpoint inhibitors treatment a clinical case by atezolizumab. *Endocrinol Diabetes Nutr (Engl Ed)* (2021) 68:363-365. doi: 10.1016/j.endien.2021.08.007

27. Tsiogka A, Jansky G, Bauer JW, Koelblinger P. Fulminant type 1 diabetes after adjuvant ipilimumab therapy in cutaneous melanoma. *Melanoma Res* (2017) 27:524-525. doi: 10.1097/CMR.0000000000000384

28. Takahashi A, Tsutsumida A, Namikawa K, Yamazaki N. Fulminant type 1 diabetes associated with nivolumab in a patient with metastatic melanoma. *Melanoma Res* (2018) 28:159-160. doi: 10.1097/CMR.0000000000000418

29. Teló, G. H., Carvalhal, G. F., Cauduro, C., Webber, V.S., Barrios, C.H., Fay, A.P. Fulminant type 1 diabetes caused by dual immune-checkpoint blockade in metastatic renal cell carcinoma. *Ann Oncol* (2017) 28:191-192. doi: 10.1093/annonc/mdw447

30. Zezza M, Kosinski C, Mekoguem C, Marino L, Chtioui H, Pitteloud N, et al. Combined immune checkpoint inhibitor therapy with nivolumab and ipilimumab causing acute-onset type 1 diabetes mellitus following a single administration: two case reports. *BMC Endocr Disord* (2019) 19:144. doi: 10.1186/s12902-019-0467-z

31. Atkins PW, Thompson DM. Combination avelumab and automilumab immunotherapy can induce diabetic ketoacidosis. *Diabetes Metab* (2018) 44:514-515. doi: 10.1016/j.diabet.2017.05.005

32. Aleksova J, Lau P, Soldatos G, McArthur G. Glucocorticoids did not reverse type 1 diabetes mellitus secondary to pembrolizumab in a patient with metastatic melanoma. *BMJ Case Rep* (2016) 2016:bcr2016217454. doi: 10.1136/bcr-2016-217454

33. Fu LL, Chen P, Wang SJ, Liu WM, Chen ZB, Chen HB, et al. Complete pathological response with diabetic ketoacidosis to the combination of sintilimab and anlotinib in an unresectable hepatocellular carcinoma patient: a case report. *Anticancer Drugs* (2022) 33:e741-e746. doi: 10.1097/CAD.0000000000001163

34. Sakai G, Saito D, Nakajima R, Hatano M, Noguchi Y, Kurihara S, et al. Intrinsic insulin secretion capacity might be preserved by discontinuing anti-programmed cell death protein 1 antibody treatment in ‘anti-programmed cell death protein 1 antibody-induced’ fulminant type 1 diabetes. *J Diabetes Investig* (2018) 9:448-449. doi: 10.1111/jdi.12662

35. Cuenca JA, Laserna A, Peyes MP, Nates J, Botz G.H. Critical Care Admission of an HIV Patient with Diabetic Ketoacidosis Secondary to Pembrolizumab. *Case Rep Crit Care* (2020) 2020:8671530. doi: 10.1155/2020/8671530

36. Peyrony O, Ellouze S, Fontaine JP, Mohamadou I, Zafrani L. Fulminant diabetes due to immune checkpoint inhibitors in the emergency department. *Am J Emerg Med* (2020) 38:408.e3-408.e4. doi: 10.1016/j.ajem.2019.158495

37. Tassone F, Colantonio I, Gamarra E, Gianotti L, Baffoni C, Magro G, et al. Nivolumab-induced fulminant type 1 diabetes (T1D): the first Italian case report with long follow-up and flash glucose monitoring. *Acta Diabetol* (2019) 56:489-490. doi: 10.1007/s00592-018-1246-4.

38. Keerty D, Das M, Julie HJ, Haynes E. Diabetic Ketoacidosis: An Adverse Reaction to Immunotherapy. *Cureus* (2020) 12:e10632. doi: 10.7759/cureus.10632

39. Maamari J, Yeung SC, Chaftari P. Diabetic ketoacidosis induced by a single dose of pembrolizumab. *Am J Emerg Med* (2019) 37:376.e1-376.e2. doi: 10.1016/j.ajem.2018.10.040

40. Ishi A, Tanaka I, Iwama S, Sakakibara T, Mastui T, Kobayashi T, et al. Efficacies of programmed cell death 1 ligand 1 blockade in non-small cell lung cancer patients with acquired resistance to prior programmed cell death 1 inhibitor and development of diabetic ketoacidosis caused by two different etiologies: a retrospective case series. *Endocr J* (2021) 68:613-620. doi: 10.1507/endocrj.EJ20-0769

41. Kapke J, Shaheen Z, Kilari D, Knudson P, Wong S. Immune Checkpoint Inhibitor-Associated Type 1 Diabetes Mellitus: Case Series, Review of the Literature, and Optimal Management. *Case Rep Oncol* (2017) 10:897-909. doi: 10.1159/000480634

42. Hong AR, Yoon JH, Kim HK, Kang H. Immune Checkpoint Inhibitor-Induced Diabetic Ketoacidosis: A Report of Four Cases and Literature Review. *Front Endocrinol (Lausanne)* (2020) 11:14. doi: 10.3389/fendo.2020.00014

43. Kyriacou A, Melson E, Chen WT, Kempegowda P. Is immune checkpoint inhibitor-associated diabetes the same as fulminant type 1 diabetes mellitus? *Clin Med (Lond*) (2020) 20:417-423. doi: 10.7861/clinmed.2020-0054.

44. Nikouline A, Brzozowski M. New DKA in a geriatric patient on immune checkpoint inhibitor therapy: a case report. *CJEM* (2021) 23:712-714. doi: 10.1007/s43678-021-00145-4

45. Kichloo A, Albosta MS, McMahon Shane, Movsesian K, Wani F, Jamal SM, et al. Pembrolizumab-Induced Diabetes Mellitus Presenting as Diabetic Ketoacidosis in a Patient with Metastatic Colonic Adenocarcinoma. *J Investig Med High Impact Case Rep* (2020) 8:2324709620951339. doi: 10.1177/2324709620951339

46. Lee S, Morgan A, Shah S, Ebeling PR. Rapid-onset diabetic ketoacidosis secondary to nivolumab therapy. *Endocrinol Diabetes Metab Case Rep* (2018) 2018:18-0021. doi: 10.1530/EDM-18-0021

47. Sankar K, Macfarlane M, Cooper O, Falk J. Pembrolizumab-Induced Diabetic Ketoacidosis: A Review of Critical Care Case. *Cureus* (2021) 13:e18983. doi: 10.7759/cureus.18983

48. Patel S, Chin V, Greenfield J R. Durvalumab-induced diabetic detoacidosis followed by hypothyroidism. *Endocrinol Diabetes Metab Case Rep* (2019) 2019:19-0098. doi: 10.1530/EDM-19-0098

49. Bastin M, Mosbah H, Carlier A, Boudifa A, Villemain A, Hartemann A, et al. Variability in clinical presentation of diabetes mellitus during anti-PD-1 immunotherapy. *Diabetes Metab* (2020) 46:406-407. doi: 10.1016/j.diabet.2019.04.005

50. Wen L, Zou XW, Chen YW, Bai XL, Liang TB. Sintilimab-induced autoimmune diabetes in a patient with the anti-tumor effect of partial regression. *Front Immunol* (2020) 11:2076. doi: 10.3389/fimmu.2020.02076

51. Yun K, Daniels G, Gold K, Mccowen K, Patel SP. Rapid onset type 1 diabetes with anti-PD-1 directed therapy. *Oncotarget* (2020) 11:2740-2746. doi: 10.18632/oncotarget.27665

52. Mellati M, Eaton KD, Barbara BW, Hagopian WA, Martins R, Palmer JP, et al. Anti–PD-1 and Anti–PDL-1 Monoclonal Antibodies Causing Type 1 Diabetes. *Diabetes Care* (2015) 38:e137-8. doi: 10.2337/dc15-0889

53. Edahiro R, Ishijima M, Kurebe H, Nishida K, Uenami T, Kanazu M, et al. Continued administration of pembrolizumab for adenocarcinoma of the lung after the onset of fulminant type 1 diabetes mellitus as an immune-related adverse effect: A case report. *Thorac Cancer* (2019) 10:1276-1279. doi: 10.1111/1759-7714.13065

54. Li SC, Zhang Y, Sun ZC, Hu J, Fang C. Anti-PD-1 pembrolizumab induced autoimmune diabetes in Chinese patient. *Medicine (Baltimore)* (2018) 97:e12907. doi: 10.1097/MD.0000000000012907

55. de Filette J, Pen JJ, Decoster L, Vissers T, Bravenboer B, Auwera AJV, et al. Immune checkpoint inhibitors and type 1 diabetes mellitus: a case report and systematic review. *Eur J Endocrinol* (2019) 181:363-374. doi: 10.1530/EJE-19-0291

55. Hakami OA, Ioana J, Ahmad S, Tun TK, Sreenan S, McDermott JH. A case of pembrolizumab-induced severe DKA and hypothyroidism in a patient with metastatic melanoma. *Endocrinol Diabetes Metab Case Rep* (2019) 2019:18-0153. doi: 10.1530/EDM-18-0153

56. Singh V, Chu Y, Gupta V, Zhao WG. A Tale of Immune-Related Adverse Events With Sequential Trials of Checkpoint Inhibitors in a Patient With Metastatic Renal Cell Carcinoma. *Cureus* (2020) 12:e8395. doi: 10.7759/cureus.8395

57. Li L, Masood A, Bari S, Yavuz S, Grosbach AB. Autoimmune Diabetes and Thyroiditis Complicating Treatment with Nivolumab. *Case Rep Oncol* (2017) 10:230-234. doi: 10.1159/000456540

58. Monteiro A, Gouveia E, Garcez D, Donato S, Diogo MB, Marques J, et al. Challenges of New Approaches in Metastatic Merkel Cell Carcinoma. *Case Rep Oncol* (2020) 13:501-507. doi: 10.1159/000507279

59. Irani AZ, Almuwais A, Gibbons H. Immune checkpoint inhibitor–induced diabetes mellitus with pembrolizumab. *BMJ Case Rep* (2022) 15:e245846. doi: 10.1136/bcr-2021-245846

60. Tohi Y, Fujimoto K, Suzuki R, Suzuki I, Kubota M, Kawakita M. Fulminant type 1 diabetes mellitus induced by pembrolizumab in a patient with urothelial carcinoma: A case report. *Urol Case Rep* (2019) 24:100849. doi: 10.1016/j.eucr.2019.100849

61. Akturk HK, Alkanani A, Zhao ZY, Yu LP, Michels AW. PD-1 Inhibitor Immune-Related Adverse Events in Patients with Preexisting Endocrine Autoimmunity. *J Clin Endocrinol Metab* (2018) 103:3589-3592. doi: 10.1210/jc.2018-01430

62. Cuenca J, Laserna A, Nates J,Gregory B. PEMBROLIZUMAB-RELATED DIABETIC KETOACIDOSIS IN THE INTENSIVE CARE UNIT: A CASE REPORT. CRITICAL CARE MEDICINE (2019) 47:537. doi: 10.1097/01.ccm.0000551288.47950.ae

63. Mengibar J, Capel I, Bonfill T, Mazarico I, Espuna L, Caixas A, et al. Simultaneous onset of type 1 diabetes mellitus and silent thyroiditis under durvalumab treatment. *Endocrinol Diabetes Metab Case Rep* (2019) 2019:19-0045. doi: 10.1530/EDM-19-0045

64. Yilmaz M, Baran A. Two different immune related adverse events occured at pancreas after nivolumab in an advanced RCC patient. *J Oncol Pharm Pract* (2022) 28:255-258. doi: 10.1177/10781552211028636

65. Tsang VHM, McGrath RT, Roderick CB, Scolyer RA, Jadrot V, Guminski AD, et al. Checkpoint Inhibitor–Associated Autoimmune Diabetes Is Distinct from Type 1 Diabetes. *J Clin Endocrinol Metab* (2019) 104:5499-5506. doi: 10.1210/jc.2019-00423

66. Galigan A, Xu W, Fourlanos S, Chiang C, Mant AM, Parente P, et al. Diabetes associated with immune checkpoint inhibition: presentation and management challenges. *Diabet Med* (2018). doi: 10.1111/dme.13762

67. Marchand L, Thivolet A, Dalle S, Chikh K, Reffet S, Vouillarmet J, et al. Diabetes mellitus induced by PD-1 and PD-L1 inhibitors: description of pancreatic endocrine and exocrine phenotype. *Acta Diabetol* (2019) 56:441-448. doi: 10.1007/s00592-018-1234-8

68. Hughes J, Vudattu N, Sznol M, Gettinger S, Kluger H, Lupsa B, et al. Precipitation of Autoimmune Diabetes with Anti-PD-1 Immunotherapy. *Diabetes Care* (2015) 38:e55-7. doi: 10.2337/dc14-2349

69. Magis Q, Caroline GM, Basire A, Loundou A, Malissen N, Troin L, et al. Diabetes and Blood Glucose Disorders Under Anti-PD1. *J Immunother* (2018) 41:232-240. doi: 10.1097/CJI.0000000000000218

70. Li W, Wang H, Chen B, Zhao S, Zhang XS, Jia KY, et al. Anti PD-1 monoclonal antibody induced autoimmune diabetes mellitus: a case report and brief review. *Transl Lung Cancer Res* (2020) 9:379-388. doi: 10.21037/tlcr.2020.03.05

71. Zaied AA, Akturk HK, Joseph RW, Lee AS. New-onset insulin-dependent diabetes due to nivolumab. *Endocrinol Diabetes Metab Case Rep* (2018) 2018:17-0174. doi: 10.1530/EDM-17-0174

72. Nishioki T, Kato M, Kataoka S, Miura K, Nagaoka T, Takahashi K. Atezolizumab-induced fulminant type 1 diabetes mellitus occurring four months after treatment cessation. *Respirol Case Rep* (2020) 8:e00685. doi: 10.1002/rcr2.685

73. Tzoulis P, Corbett RW, Ponnampalam S, Baker E, Heaton D, Doulgeraki T, et al. Nivolumab-induced fulminant diabetic ketoacidosis followed by thyroiditis. *Endocrinol Diabetes Metab Case Rep* (2018) 2018:18-0111. doi: 10.1530/EDM-18-0111

74. Tang Y, Zhao Z, Wang XF, Zuo W, Zhang B, Yuan T, et al. A case of pembrolizumab-induced fulminant Type 1 diabetes mellitus in breast cancer. *Immunotherapy* (2021) 13:483-489. doi: 10.2217/imt-2020-0222
