# Supplementary material for: Combined diabetic ketoacidosis and hyperosmolar hyperglycemic state in type 1 diabetes mellitus induced by immune checkpoint inhibitors: Underrecognized and underreported emergency in ICIs-DM
Source: Front Endocrinol (Lausanne). 2023 Jan 4;13:1084441. doi: 10.3389/fendo.2022.1084441 (PMC9846077; doi:10.3389/fendo.2022.1084441)
Supplement: Supplementary file 3 [file Table_3.docx]

**S Table 3 |** The clinical characteristics of reported cases with ICIs-induced combined DKA-HHS

|  | Case 1 | Case 2 | Case 3 | Case 4 | Case 5 | Case 6 | Case 7 | Case 8 | Case 9 |
| --- | --- | --- | --- | --- | --- | --- | --- | --- | --- |
| Age/Sex | 67/M | 71/M | 74/F | 49/M | 61/M | 72/F | 75/M | 69/M | 67/M |
| Neoplasia | testicular lymphoma | Non-small-cell lung cancer | Non-small-cell lung cancer | Renal cell carcinoma | Metastatic melanoma | Renal cell carcinoma | Non-small-cell lung cancer | Non-small-cell lung cancer | urothelial cancer |
| Relevant background | Transient hyperglycemia related to steroids as part of the chemotherapy protocal | Obesity (BMI 32kg/m^2^), hypertension, dyslipidaemia, T_2_DM history of mother | Arterial hypertension;  hypercholesterolaemia | Dandy-Walker syndrome;  epilepsy | - | - | COPD; hypertension; dyslipidemia; pulmonary embolism; benign prostatic hyperplasia | T_2_DM | - |
| Prior cancer-related therapy | Rituximab+cyclophosphamide+hydroxydaunorubicin+oncovin+prednison; rituximab+etoposide+steroid+Ara-C+cisplatin;  Ifosfamide+carboplatin+etoposide | Cisplatin +pemetrexed | Pemetrexed | Sunitinib | High-dose prednisone because of rash | Chemotherapy (no detail) | Chemo-radiation therapy (carboplatin+paclitaxel) | Carboplatin+pemetrexed | - |
| Immunotherapy | Nivolumab | Nivolumab | Nivolumab | Nivolumab | Nivolumab+ipilimumab | Pembrolizumab | Durvalumab | Pembrolizumab | Atezolizumab |
| No. cycles before diabetes onset | 7 | 14 | 2 | 21 | 3 | 6 | 2 | 4 | 9 |
| Diagnosis of diabetes | ADM | DM | T1DM | T1DM | FD | DM | ADM | T1DM | T1DM |
| Clinical symptoms  at onset of diabetes | Vomiting, abdominal pain and confusion | Polyuria, acute renal failure | Polyuria, polydipsia, weight loss (5kg), confusion, asthenia, dehydration, hypotension, and Kussmaul respiratory pattern | Severe asthenia, somnolence, polyuria, polydipsia, and weight loss | Nausea, vomiting, polyuria, malaise, decreased PO intake, and dizziness | Altered mental status, and vomiting | Dyspnea, generalized weakness, dizziness, dysphagia, nausea/vomiting, and diarrhea | nausea, vomiting, polyuria, and polydipsia | General weakness and polydipsia |
| Other irAEs | - | - | - | - | Maculopapular rash | - | - | - | - |
| pH | 7.132 | NA | 7.07 | 7.27 | 7.14 | NA | 6.99 | 6.95 | 7.166 |
| Serum sodium | 132 | NA | 142^#^ | 138 | 127 | 124 | 122 | NA | NA |
| Serum osmolarity (280-290) mOsmol/kg H_2_O | 336^+^ | 322^+^ | 389^+^ | 320^++^ | 321^++^ | 323^++^ | 329^++^ | 342^+^ | 341^+^ |
| Ketones | Positive | Negative | Positive | Positive | Positive | Positive | Positive | Positive | Positive |
| Bicarbonate | 5.6mmol/l | NA | 7.8mmol/l | 17.8mmol/l | ＜10mmol/l | NA | 2mmol/l | 3mmol/l | 13.2mmol/l |
| Anion gap | NA | NA | 41mmol/l | 21mmol/l | ＞31mmol/l | 36mEq/l | 33mmol/l | 39mmol/l | NA |
| DKA | Yes | No | Yes | Yes | Yes | Yes | Yes | Yes | Yes |
| C-peptide levels | Undetectable  (<0.02 ng/mL) | 2ug/l ^*^  (0.01-4.2) | 0.2 ng/mL  (1.1-4.4) | 0.079 nmol/l (0.4-1.5) | Undetectable (<0.1ng/ml) | NA | NA | Undetectable (<0.1mg/ml) | 0.01 ng/mL  (1.1-4.4) |
| Blood glucose mg/dL | 1008 | 792 | 1060 | 801 | 1211 | 1342 | 1532 | 907 | 530 |
| HbA1c (%) | 8 | 13.7 | 8.7 | 10.9 | 6.9 | 10.1 | 7.5 | 9.2 | 9.8 |
| Autoantibodies positivity | - | - | GAD | - | - | - | GAD | GAD | - |
| HLA genotype | *DRB1* | *DRB1*04:02;*  *DQB1*03:02* | *DRB1*04* | NA | NA | NA | NA | NA | NA |
| Pancreatic enzyme elevation | - | Lipase | Lipase;  Amylase | NA | Lipase | NA | NA | NA | NA |
| Acute renal failure | Yes | Yes | Yes | Yes | Yes | NA | Yes | Yes | - |
| Suspected infection | NA | NA | - | - | NA | NA | Yes | - | NA |
| Clinical outcome  and follow-up | Insulin therapy;  Resume nivolumab | Insulin therapy | Insulin therapy;  Continue nivolumab | Insulin therapy;  Continue nivolumab | Insulin therapy | Insulin therapy;  Continue pembrolizumab | Insulin therapy;  Continue durvalumab | Combined insulin therapy with metformin;  Resume pembrolizumab | Insulin therapy;  Continue atezolizumab |

*T2DM, type 2 diabetes mellitus; BMI, body mass index; COPD,chronic obstructive pulmonary disease; ADM, autoimmune diabetes mellitus; NA, not available; -, negative; GAD, glutamic acid decarboxylase.*

*^*^: The patient still had a persistent insulin secretion at the time of diagnosis.*

*^+^: The values were copied from reported papers.*

*^++^: This table included the 4 additional cases (case 4, 5, 6 and 7) calculated by our research group, the effective serum osmolality was calculated by 2*[measured serum Na+ (mEq/L)] + glucose (mg/dl)/18 referenced from ref.（8）.*

*^#^: The value was showed in corrected sodium from the reported paper.*
